# Towards Predictive Synthesis of Inorganic Materials Using Network Science

**DOI:** 10.3389/fchem.2021.798838

**Published:** 2021-12-21

**Authors:** Alex Aziz, Javier Carrasco

**Affiliations:** Centre for Cooperative Research on Alternative Energies (CIC energiGUNE), Basque Research and Technology Alliance (BRTA), Vitoria-Gasteiz, Spain

**Keywords:** synthesis by design, materials synthesis networks, inorganic material design, network science, Inorganic synthesis

## Abstract

Accelerating materials discovery is the cornerstone of modern technological competitiveness. Yet, the inorganic synthesis of new compounds is often an important bottleneck in this quest. Well-established quantum chemistry and experimental synthesis methods combined with consolidated network science approaches might provide revolutionary knowledge to tackle this challenge. Recent pioneering studies in this direction have shown that the topological analysis of material networks hold great potential to effectively explore the synthesizability of inorganic compounds. In this Perspective we discuss the most exciting work in this area, in particular emerging new physicochemical insights and general concepts on how network science can significantly help reduce the timescales required to discover new materials and find synthetic routes for their fabrication. We also provide a perspective on outstanding problems, challenges and open questions.

## Introduction

Advanced materials are key enablers across many industries aimed at addressing the global challenges of economic security, renewable and sustainable energy, and human welfare. Innovation in these fields often requires searching for new materials or optimizing existing ones. The traditional materials discovery approach is to focus on archetypal compounds in which a desirable property was first observed. This approach involves trial-and-error chemical exploration, which usually has high demands in terms of synthesis times and costs. Therefore, accelerating the pace of discovery of new materials is essential to achieving global competitiveness in the 21^st^ century. Computational modelling has emerged as a powerful complementary tool in accelerating the process of materials discovery. Thanks to the proven predictive power of quantum chemistry methods, together with the spectacular growth of computational resources, computer modelling is nowadays able to bring valuable insights in understanding the structure, properties, and function of technological materials. In particular, high-throughput screening of materials databases using first-principles simulation approaches have demonstrated a successful track record of guiding advances in materials science (Jain et al., 2016), including areas as diverse as heterogeneous catalysis (Greeley et al., 2002), thermoelectricity (Carrete et al., 2014), and energy storage (Van der Ven et al., 2020). With an increase in computer resources and given computational modelling is progressively being implemented in synergy with experiments, this trend is only likely to grow. However, computational simulations in particular *ab initio* molecular dynamics are computationally demanding, energy intensive and risk being repeated multiple times by various groups investigating similar materials.

An emerging alternative to traditional physical-based approaches is data-driven modelling (Agrawal and Choudhary, 2016; Jennings et al., 2019; Noh et al., 2020; Lombardo et al., 2021). As a matter of fact, recent trends in Big Data have raised hopes for a new kind of paradigm to model complex systems with a large number of strongly interacting elements. And autonomous decision-making materials discovery schemes to guide experimental campaigns are starting to emerge (Montoya et al., 2020; Stach et al., 2021; Szymanski et al., 2021). Data science may indeed help to answer many fundamental research questions, especially as more and more data becomes accessible (Hill et al., 2016). This is evident from the rise in number and quality of computational materials databases and related informatics such as the Materials Project (materialsproject.org), AFLOWLIB (aflowlib.org), NoMaD (nomad-coe.eu) and the Open Quantum Materials Database (OQMD) (oqmd.org) that complement existing experimental data sets such as the Inorganic Crystal Structure Database (ICSD) (icsd.products.fiz-karlsruhe.de), NIST Materials Data Repository (nist.gov), or the Pauling File (paulingfile.com). However, data science alone cannot develop fundamental research questions by itself. Collecting data and then identifying new patterns has the potential risk of ending up with spurious correlations, without understanding the underlying causal relationships. Indeed, this applies to all data driven approaches, and care must be taken to benchmark and verify datasets with experiment.

From a theoretical viewpoint, materials discovery faces a two-fold major paradigm. On the one hand, the identification of thermodynamically stable compounds, also referred to as a structure prediction problem. And on the other, synthesizability, which typically involves evaluating metastable lifetimes and reaction energies. Thanks to a number of methodological developments in the last 20 years, reliable structure prediction can nowadays be efficiently performed without any prior knowledge or assumptions about the system (Goedecker, 2004; Oganov et al., 2019; Tong et al., 2019). The ability of these methods to predict not only the ground states, but also low-energy metastable structures is indeed leading to the identification of an increasing number of new virtual materials. Thermodynamic considerations narrow down the chemical space for where experimentalists should look (Szczypinski et al., 2021) and indicate the synthesis probability of stable and metastable structures in a first rung approach (Aykol et al., 2018). Yet, the problem of synthesizability remains. As a consequence, the continuous proposition of new virtual materials with optimal properties is often seen from experimentalists as a dreamland of unachievable real materials. Without an efficient way to assess actual synthetic routes towards novel stable compounds, theoretical materials discovery is severely hindered. The problem of synthesizability is exceptionally hard to solve because as it needs to be addressed in a holistic manner. In principle, predicting feasible synthetic routes for a new material requires not only finding the lowest energy structures of candidate reactants and products, but also proposing plausible multi-step reaction mechanisms (including possible metastable compounds) and computing transition state structures. Headway is being made and new strategies are being proposed to incorporate the dynamics of these complex chemical spaces. One such strategy is the high-throughput analysis of possible reaction pathways to target a specific inorganic crystal phase by through reaction energies of reactants, the number of competing phases and approximated nucleation barriers, at each step, thereby identifying preferential synthesis routes (Aykol et al., 2021). An alternative strategy employs the use of neural networks to generate synthesis predictions for inorganic materials by mining the scientific literature (Kim et al., 2020). This approach would benefit from the multitude of synthesis data from unsuccessful experimental attempts, if such data was to be made publicly available, as suggested by Kovnir, 2021. However, the use of experimental synthesis data in data driven approaches has been shown to have anthropogenic biases in the choice of reagents and reaction conditions that may ultimately lead to skewed networks (Jia et al., 2019). In a computational approach the consideration of both thermodynamics and kinetics along reaction pathways could target the synthesis of any hypothetical material with properties of interest, but leads to the exploration of large chemical spaces and requires the use of sophisticated and computationally demanding methods.

In this perspective we focus on a strategy based on the application of network science (Barabási, 2016) that is starting to gain momentum, using the power of network-based representations and topological analysis to examine solid-state chemical reactivity for materials discovery, specifically a graph based approach to mapping the thermodynamic relationships between different materials. This bridge between the discovery of new virtual materials and the simultaneous identification of likely synthetic routes could guide experiments and accelerate materials design and development.

## Materials Networks

**FIGURE 1:**
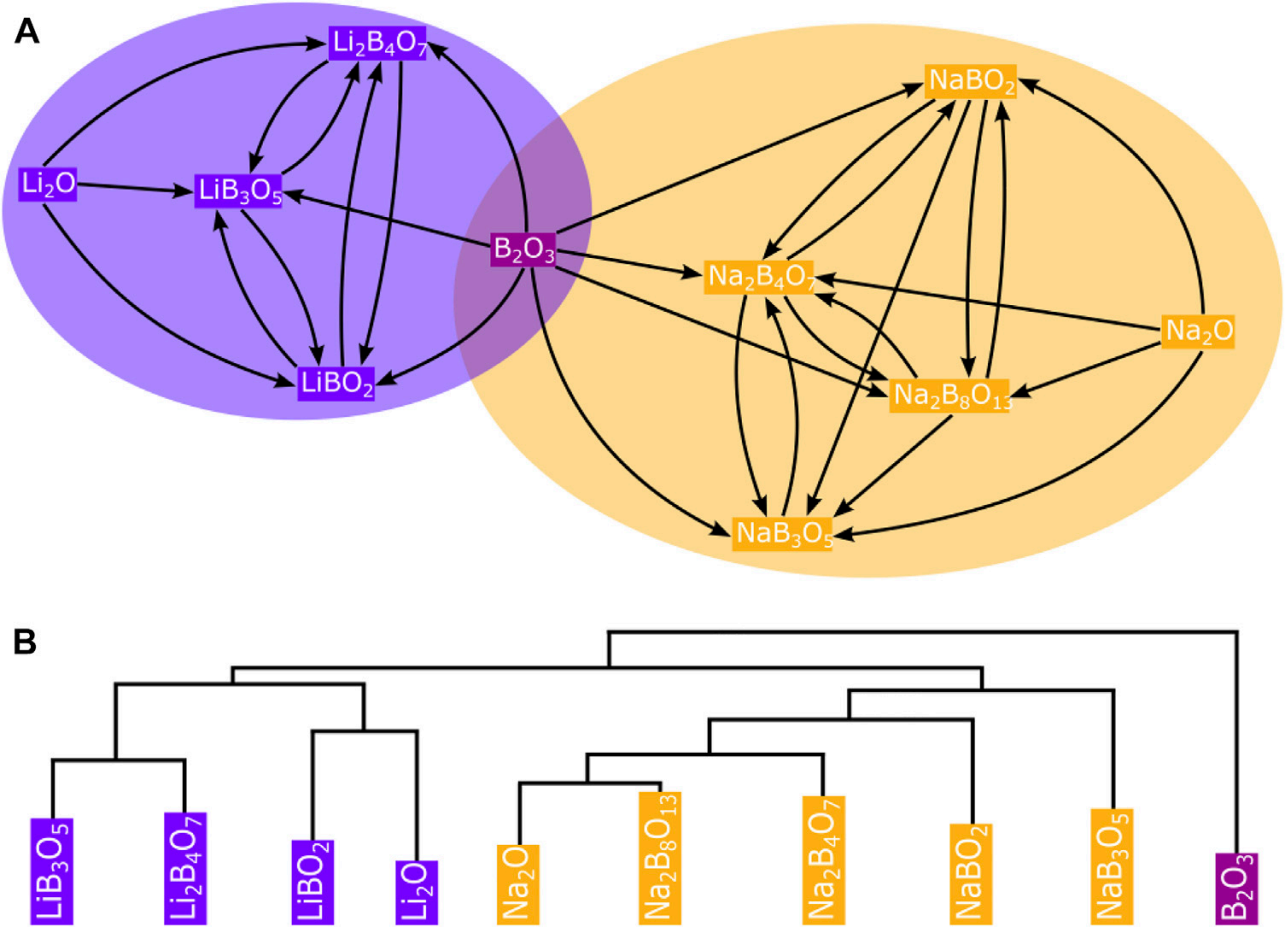
A network of A + B → C solid-state reactions (see Supplementary Table S1 in the Electronic Supplementary Information for details) is used here with the clique percolation method implemented in CFinder (Adamcsek et al., 2006) to automatically identify only one common node (B_2_O_3_) between the two communities in the Li-B-O-Na chemical space **(A)**. Additionally, the dendrogram generated by the Girvan-Newman algorithm in **(B)** using Networkx helps to systematically reproduce the modules built into the network (Hagberg et al., 2008).

Networks are very simple models, yet extremely useful to represent complex systems, where the components of the graph system are represented by nodes and their interactions by links or edges. These links can be undirected (lines) or directed (arrows), depending on the system’s nature. For example, a molecular chemical reaction network can be represented as a directional connected graph. The reactants, traverse a complex chemical space along reaction pathways (links) that are governed by kinetics, through intermediates (nodes) breaking and forming bonds, before finally reaching the desired products. In contrast, in the crystalline network of a solid, the nodes represent atoms and the links (bonds) are undirected (Blatov et al., 2019, 2021). What makes networks useful is that their interaction structure (i.e., the network’s topology) accounts for their systemic properties and, therefore, topological analysis can lead to applicable, impactful outcomes. Topological characterization of networks includes centrality analysis by computing average degree and degree distributions (the degree is the number of links a node has to other nodes) as well as other more complex characteristics such as clustering coefficients, betweenness, or hierarchy (Barabási, 2016). Figure 1 illustrates how some of these topological characterizations can be useful, with a simple materials network example built up from experimental thermochemistry data, to analyse inorganic reactivity and identify common nodes in large chemical spaces.

With the availability of computational materials databases and the development of network theory we now have the underlying data and technical know-how to utilize network science in material discovery. To date there have been a few representative studies modelling chemical spaces using networks that have predominately been focused on fragment-based drug discovery and ligand-based screening of organic molecules (Tanaka et al., 2009; Kunimoto et al., 2017). In deciphering reaction mechanisms a novel approach employs the PageRank algorithm as a collective variable to graph the possible molecular topologies along a specific reaction pathway (Zhou et al., 2019). Taking a more general approach, the pioneering work by Gothard et al., 2012 demonstrated that the construction of a directional network from organic reactions reported in the literature can predict sequential synthesis steps using specific chemical filters including functional groups and synthesis conditions in a one-pot approach. Only recently, has this approach gained the attention of the inorganic research community; from both a pure crystal structure prediction perspective (Ahnert et al., 2017) and in the consideration of synthesizability (Aykol et al., 2019; Hegde et al., 2020; Blau et al., 2021). From a chemistry, and materials science point of view network representations are indeed a good approach to tackle synthesizability for the following reasons:

(i) Chemical reaction spaces are generally very high dimensional, the need to reduce this dimensionality often results in a loss of information. Network representations avoid this issue as there is no need for the construction of a coordinate system or for any form of dimensionally reduction. Networks are a natural representation of chemical reactions (Choudhury et al., 2020).
(ii) Network science provides an intuitive conceptual framework to statistically analyse many aspects of reaction spaces and synthesis strategies, with many meaningful descriptors (e.g., hubs, communities, hierarchy, and betweenness, among many others) (Barabási, 2016).
(iii) The rapidly expanding study of complex networks across a wide range of disciplines has given rise to a large arsenal of efficient algorithms and mathematical approaches to quantify network properties and interpret their characteristics. This development in network science paves the way to apply these tools to synthesizability.

## Examples of Topological Analysis of Material Networks

Hegde and coworkers have recently developed a unidirectional materials network encoding the thermodynamic stability (at T = 0 K) in the OQMD database (Hegde et al., 2020). The network comprises of ∼21,300 nodes (inorganic compounds) with each node able to connect to ∼3,850 edges, which represents the number of two phase equilibria (thermodynamic equilibrium) between phases, and highlights the dense nature of the network. The comprehensive mapping of this materials network allows a top down approach to tackling material stability, as a material’s nobility is measured as a function of the count (or number of edges) of materials it has no reactivity against. As more data is added this network has the scope to evolve and verify itself. Holes in the network may identify materials yet to be discovered, and subsequent topological analysis may offer an approach to realize them starting with adjacent structures in the network. Their discovery and synthesis will lead to the validation of the network model and wide scale acceptance of network theory as a strategy in materials discovery. In essence similar to the gaps or holes in the periodic table predicted by Mendeleev in 1869, with the first such hole filled with the discovery of gallium in 1875 validating Mendeleev’s periodic law.

The progressive development of network analysis may well guide experimentalists to decipher which stable predicted structures can indeed be synthesised. As an alternative to determining synthesizability from thermodynamic considerations, a novel time analysis approach combined with machine learning has given a glimpse of how networks could be utilized in this direction (Aykol et al., 2019). To reduce the size of the network a subsample is taken, considering only materials that share an edge with at least one physically stable material in the same chemical space. An analysis of the network reveals some interesting insights; the network is determined to be scale-free: some nodes have a significantly larger number of edges and are thus referred to as hubs. This has two implications; materials missing from the database will not hinder the discovery of others, but missing hubs imply materials yet to be discovered and identifying new hubs will accelerate the discovery in those spaces. Using a machine-learning model based on certain network properties of materials Aykol et al. (2019) determine the likelihood of a predicted material in the network to actually be synthesised but do not give an insight on their synthesis pathway. In this respect the combination of a series of networks seems natural. First, a directional network approach to determine the probability of synthesis of a new material. Subsequently, a directed reaction network approach to identify low-cost and plausible reaction pathways for its fabrication. Ideally, such an approach would employ optimized pathfinding algorithms similar to those in car navigation systems where one starts at point A (the reactant) and finishes at point B (the product) whilst choosing the quickest routes dependent on the traffic (kinetics), but also considering intermediates, radicals, and ions, which will have different stabilities dependent on their phase and synthesis conditions, all whilst maintaining stoichiometric constraints. This complexity is a significant challenge that limits the size of such a reaction network (Unsleber and Reiher, 2020). In this regard neural networks have shown promise in navigating the huge network space in organic molecular systems. Recently, a three layered neural network has been able to uncover retrosynthetic routes through the use of Monte Carlo tree search algorithms (Segler et al., 2018) based on reactions found in the Reaxys database, and we refer the reader to a recent review on machine-learning methods for more information (Meuwly, 2021). Compared to molecular synthesis, inorganic synthesis prediction is more complex, given the sheer number of elements, metastability and the possibility of new unchartered materials. However, materials networks have made progress, interdependencies between materials have now been implemented in a directional network that estimates the cost of going from reactant to product ensuring stoichiometry is preserved along the path (Blau et al., 2021). To ensure stoichiometry the network space is continually expanded to ensure all the costs of producing or removing the additional reactants required in the network are accounted for. The network determines the cost solely on thermodynamic considerations, but as databases expand, other parameters such as kinetics, experimental reaction yields, or the cost of precursors and their toxicity could also be included. Indeed this has been demonstrated in subsequent work expanding their network to include local chemical potential (Todd et al., 2021). The success of the network is illustrated by its ability to identify both proposed and novel-pathways in the formation of lithium ethylene dicarbonate that forms at the solid electrolyte interphase at the anode of lithium ion batteries. Despite 6,000 species being needed with the analysis of over 4.5 million reactions the complete network was deduced on a laptop in less than a day, highlighting the power of such a tool. The 5 “shortest pathways” or most likely synthesis routes are identified, two of which have previously been purported in the literature (Blau et al., 2021). The omission of kinetics in the network may lead to certain reaction pathways being omitted or identified but unfeasible. One way to incorporate kinetics is their subsequent manual consideration once a set of lowest cost pathways is identified. This approach is employed to determine whether lithium ethylene monocarbonate or dicarbonate forms at the solid electrolyte interphase (Xie et al., 2021). After construction of the graph reaction network and elimination of duplicate pathways, the predominant pathways are analysed, leading to the conclusion that paths without the presence of water are kinetically unfeasible due to large energy barriers. The requirement of water in the reaction pathway limits the formation of lithium ethylene monocarbonate and also suggests varying the water content at the interface could control the ratio of formation of lithium ethylene monocarbonate or dicarbonate. Such an insight is clearly invaluable for experiments.

**FIGURE 2:**
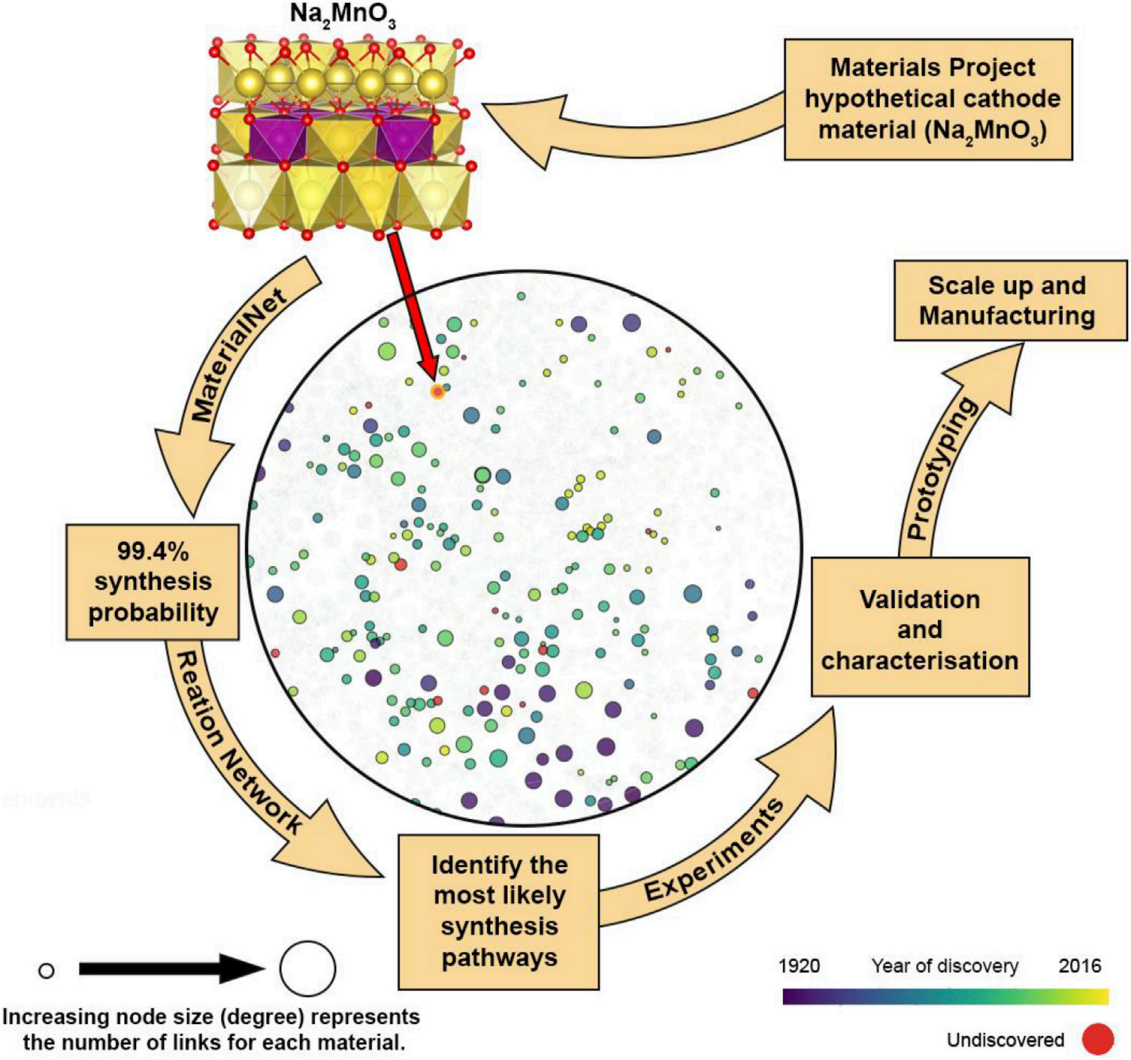
Visualization of a local network for the hypothetical (undiscovered) material Na_2_MnO_3_ (Gao et al., 2019) generated using the MaterialNet web application (Choudhury et al., 2020) and expected to have a 99.4% probability of synthesis. To illustrate the local network environment Na and NaO derived materials are also added to the chemical subspace. A reaction network (McDermott et al., 2021) could then be employed to identify the most likely synthesis pathways. In the structural model Na atoms are shown in yellow, Mn atoms in purple and O atoms in red.

A somewhat simpler graph-based network that considers only the thermodynamics of solid-state reactions built up from the Material Project database and utilizes machine learning has shown promise in predicting complex reaction pathways (McDermott et al., 2021). Again, only taking into account thermodynamic considerations both negative and positive free energies are mapped as positive costs using the softplus function (Dugas et al., 2001). This is a standard practise to ensure standard pathfinding algorithms can be used. Without kinetic considerations this network is sufficient to predict the complex reaction pathways reported in the literature for YMnO_3_, Y_2_Mn_2_O_7_, Fe_2_SiS_4,_ and YBa_2_Cu_3_O_6.5_. Derived reaction routes may well include hypothetical intermediates; in the case of YMnO_3_, the hypothetical compound Li_3_MnO_3_ is identified and ignored in the study. In the case of Fe_2_SiS_4_, a system of only three elements less stringent constraints on metastability above the hull of 0.5 eV per atom can be incorporated. This highlights that even with relatively straightforward thermodynamic network models trade-offs are still required. Indeed, the maximum number of reaction pathways (pathfinding processes) and reaction combinations in reaching the final product are also of consideration and are set as parameters in the network. The power of this network model is demonstrated by the possibility of “synthesis by design”, with the suggested synthesis routes for a hypothetical material MgMo_3_(PO_4_)_3_O that has been predicted to have superior Mg^2+^ mobility (Rong et al., 2017). It is now also possible to visualize certain available database online (maps.matr.io) through the MaterialNet interactive map (Choudhury et al., 2020). In Figure 2 we take Na_2_MnO_3_, an undiscovered hypothetical material reported in the literature (Gao et al., 2019) and use the Materials Stability Network to identify other similar materials in its chemical space and find its expected synthesis probability to be 99.4%. The next step in this top down approach would be to identify possible synthesis pathways followed by experimental validation. The identification of possible synthesis routes would help experimentalists reduce the number of reactions pathways to consider even if ultimately the network failed to predict the optimal reaction pathway.

## Discussion

Advances in network models complemented with the recent explosion of materials databases presents an opportunity to develop a new pioneering research area in materials discovery and synthesis. Holes or gaps in networks may help identify materials yet to be discovered and predictive synthesis routes identified. To ensure the network representations are an accurate representation of the chemical space, one must ensure the data is complete, accurate and with no inherent bias. From a computational perspective network models are highly dependent on their original data and the difficulty in standard density functional theory approaches in dealing with correlated systems raises questions on the validity of the *f*-block (and to a lesser extent later *d*-block) thermodynamic data, and how to accurately include them in the network. From an experimental perspective anthropogenic biases in the choice of reagents and reaction conditions in experimental synthesis may lead to skewed networks (Jia et al., 2019). The immense chemical space; for example, 10^10^ combinations of possible materials for the quaternary compounds formed from the first 103 elements are proposed (Davies et al., 2016), leads to a trade-off between network size and detailed synthesis prediction. While, the complete Materials Project network can be analysed to predict the probability of a hypothetical structure being synthesized, a much more detailed network is needed to suggest a synthesis pathway, especially if molecular precursor reactions are incorporated. Further development of materials databases and/or machine learning approaches will also be needed to incorporate kinetic costs or take into account other considerations such as reaction yields, toxicity, and configurational disorder or to predict the space group of a material. Whilst the omission of kinetics and other considerations, may lead to an incorrect hierarchy of predicted pathways, the number of synthesis pathways trialled could be dramatically reduced, maximising an experimentalist`s research time. As this research area evolves it will no doubt be an extremely powerful technique to add to the arsenal available to the material-science community.

## Data Availability

The original contributions presented in the study are included in the article/Supplementary Material, further inquiries can be directed to the corresponding author.
