# Supplementary material for: Towards Predictive Synthesis of Inorganic Materials Using Network Science
Source: Front Chem. 2021 Dec 21;9:798838. doi: 10.3389/fchem.2021.798838 (PMC8724131; doi:10.3389/fchem.2021.798838)
Supplement: Supplementary file 1 [file DataSheet1.PDF]

Electronic Supplementary Information

Towards predictive synthesis of inorganic materials using network science

Alex Aziz and Javier Carrasco [*]

Centre for Cooperative Research on Alternative Energies (CIC energiGUNE), Basque Research and Technology Alliance (BRTA), Alava Technology Park, Albert Einstein 48, 01510 Vitoria-Gasteiz, Spain

[*]jcarrasco@cicenergigune.com

Table S1. List of solid-state reactions considered to build the reaction network shown in Figure 1. The enthalpy of each reaction at standard conditions ($\Delta_r H°_{solid}$) has been calculated using the experimental thermochemistry data reported in Table S2. Negative $\Delta_r H°_{solid}$ indicates that the reaction is exothermic.

**Reaction**	**$\Delta_r H°_{solid}$ (kJ/mol)**
$Li_2O + Li_2B_4O_7 \rightarrow 4\ LiBO_2$	–115.88
$Li_2O + 2\ B_2O_3 \rightarrow Li_2B_4O_7$	–216.54
$Li_2O + 3\ B_2O_3 \rightarrow 2\ LiB_3O_5$	–252.56
$½\ Li_2O + ½\ B_2O_3 \rightarrow LiBO_2$	–83.10
$¼\ Li_2O + LiB_3O_5 \rightarrow ¾\ Li_2B_4O_7$	–36.12
$Li_2O + LiB_3O_5 \rightarrow 3\ LiBO_2$	–123.03
$2\ LiBO_2 + B_2O_3 \rightarrow Li_2B_4O_7$	–50.33
$LiBO_2 + LiB_3O_5 \rightarrow Li_2B_4O_7$	–7.15
$LiBO_2 + B_2O_3 \rightarrow LiB_3O_5$	–43.18
$½\ Li_2B_4O_7 + ½\ B_2O_3 \rightarrow LiB_3O_5$	–18.01
$Na_2O + 2\ B_2O_3 \rightarrow Na_2B_4O_7$	–311.77
$Na_2O + 4\ B_2O_3 \rightarrow Na_2B_8O_{13}$	–390.82
$Na_2O + 3\ B_2O_3 \rightarrow 2\ NaB_3O_5$	–359.72
$½\ Na_2O + ½\ B_2O_3 \rightarrow NaBO_2$	–129.96
$Na_2O + Na_2B_4O_7 \rightarrow 4\ NaBO_2$	–208.07
$3\ Na_2O + Na_2B_8O_{13} \rightarrow 8\ NaBO_2$	–648.86
$Na_2O + NaB_3O_5 \rightarrow 3\ NaBO_2$	–210.02
$½\ Na_2O + ½\ B_2O_3 \rightarrow NaBO_2$	–129.96
$Na_2O + 3\ Na_2B_8O_{13} \rightarrow 8\ NaB_3O_5$	–266.42
$Na_2O + 4\ NaB_3O_5 \rightarrow 3\ Na_2B_4O_7$	–215.87
$½\ Na_2O + ½\ Na_2B_8O_{13} \rightarrow Na_2B_4O_7$	–116.36
$2\ NaBO_2 + B_2O_3 \rightarrow Na_2B_4O_7$	–51.85
$NaBO_2 + B_2O_3 \rightarrow NaB_3O_5$	–49.90
$4\ NaBO_2 + Na_2B_8O_{13} \rightarrow 3\ Na_2B_4O_7$	–24.66
$NaBO_2 + NaB_3O_5 \rightarrow Na_2B_4O_7$	–1.95
$2\ NaBO_2 + 3\ B_2O_3 \rightarrow Na_2B_8O_{13}$	–130.90
$NaBO_2 + Na_2B_8O_{13} \rightarrow 3\ NaB_3O_5$	–18.80
$2\ NaB_3O_5 + B_2O3 \rightarrow Na_2B_8O_{13}$	–31.10
$Na_2B_4O_7 + B_2O_3 \rightarrow 2\ NaB_3O_5$	–47.95
$Na_2B_4O_7 + B_2O_3 \rightarrow Na_2B_8O_{13}$	–79.05
$Na_2B_4O_7 + Na_2B_8O_{13} \rightarrow 4\ NaB_3O_5$	–16.85

Table S2. Enthalpy of formation of solid at standard conditions ($\Delta_f H°_{solid}$).

**Formula**	**$\Delta_f H°_{solid}$ (kJ/mol)**	**Reference**
Li_2O	–598.73	Chase, 1998
$LiBO_2$	–1019.22	Chase, 1998
LiB_3O_5	–2335.90	Kubaschewski et al., 1993
$Li_2B_4O_7$	–3362.27	Chase, 1998
Na_2O	–417.98	Chase, 1998
$NaBO_2$	–975.70	Kubaschewski et al., 1993
NaB_3O_5	–2299.10	Kubaschewski et al., 1993
$Na_2B_4O_7$	–3276.75	Chase, 1998
$Na_2B_8O_{13}$	–5902.80	Kubaschewski et al., 1993
B_2O_3	–1273.50	Cox et al., 1984

REFERENCES

Chase, M.W., Jr., NIST-JANAF Themochemical Tables, Fourth Edition, J. Phys. Chem. Ref. Data, Monograph 9, 1998, 1-1951.

Cox, J.D., Wagman, D.D., Medvedev, V.A., CODATA Key Values for Thermodynamics, Hemisphere Publishing Corp., New York, 1984, 1.

Kubaschewski, O., Alcock, C., Spencer, P., Materials Thermochemistry, 6th ed., Oxford, Pergamom Press, 1993.
